# NK Cells Augment Oncolytic Adenovirus Cytotoxicity in Ovarian Cancer

**DOI:** 10.1016/j.omto.2020.02.001

**Published:** 2020-02-15

**Authors:** Elaine Y.L. Leung, Darren P. Ennis, Philippa R. Kennedy, Christopher Hansell, Suzanne Dowson, Malcolm Farquharson, Pavlina Spiliopoulou, Jaya Nautiyal, Sophie McNamara, Leo M. Carlin, Kerry Fisher, Daniel M. Davis, Gerard Graham, Iain A. McNeish

**Affiliations:** 1 Institute of Cancer Sciences, University of Glasgow, Glasgow, UK; 2 Institute of Infection, Inflammation and Immunity, University of Glasgow, Glasgow, UK; 3 Ovarian Cancer Action Research Centre and Division of Cancer, Department of Surgery and Cancer, Imperial College, London, UK; 4 Manchester Collaborative Centre for Inflammation Research, University of Manchester, Manchester, UK; 5 Cancer Research UK Beatson Institute, Glasgow, UK; 6 PsiOxus Therapeutics, Oxford, UK

**Keywords:** Ovarian cancer, Oncolytic virus, Adenovirus, NK cell, DNAM-1, TIGIT

## Abstract

Oncolytic viruses (OVs) can trigger profound innate and adaptive immune responses, which have the potential both to potentiate and reduce the activity of OVs. Natural killer (NK) cells can mediate potent anti-viral and anti-tumoral responses, but there are no data on the role of NK cells in oncolytic adenovirus activity. Here, we have used two different oncolytic adenoviruses—the Ad5 E1A CR2-deletion mutant *dl*922-947 (group C) and the chimeric Ad3/Ad11p mutant enadenotucirev (group B)—to investigate the effect of NK cells on overall anti-cancer efficacy in ovarian cancer. Because human adenoviruses do not replicate in murine cells, we utilized primary human NK cells from peripheral blood and ovarian cancer ascites. Our results show that *dl*922-947 and enadenotucirev do not infect NK cells, but induce contact-dependent activation and anti-cancer cytotoxicity against adenovirus-infected ovarian cancer cells. Moreover, manipulation of NK receptors DNAM-1 (DNAX accessory molecule-1) and TIGIT (T cell immunoreceptor with Ig and ITIM domains) significantly influences NK cytotoxicity against adenovirus-infected cells. Together, these results indicate that NK cells act to increase the activity of oncolytic adenovirus in ovarian cancer and suggest that strategies to augment NK activity further via the blockade of inhibitory NK receptor TIGIT could enhance therapeutic potential of OVs.

## Introduction

Oncolytic viruses (OVs) infect malignant cells and replicate selectively within them. This selective replication induces direct cytotoxicity and also triggers profound innate and adaptive immune responses,^1^, ^2^, ^3^ which have the potential both to potentiate and reduce the anti-cancer activity of the OVs.^4^

Oncolytic adenoviruses have shown potential as cancer therapeutics in multiple pre-clinical tumor models,^5^, ^6^, ^7^ and the first oncolytic virus licensed for routine clinical practice was an adenovirus.^8^ Two oncolytic adenoviruses were evaluated in this work: the E1A CR2-deleted Ad5 mutant *dl*922-947, which we have previously shown has significant efficacy in ovarian cancer models,^9^^,^^10^ and the chimeric Ad3/Ad11p mutant enadenotucirev, which was generated by directed evolution^11^ and is currently being tested in early phase clinical trials in ovarian (ClinicalTrials.gov: NCT02053220)^12^ and other cancers (ClinicalTrials.gov: NCT02028117).

Natural killer (NK) cells are a group of effector innate immune cells that can mediate potent anti-viral and anti-tumoral responses (reviewed in Jos and Altfeld^13^ and Marcus et al.^14^). In the context of oncolytic virotherapy, NK cells activated by the viral infection could potentially lead to efficient elimination of the infected cancer cells, but rapid clearance of virus-infected cells may also prevent the spread of OVs and reduce overall activity. Previous studies suggested that NK cells are activated by oncolytic reovirus treatment in patients^15^ and are necessary for successful maraba virus therapy in murine models.^16^ By contrast, NK cells may limit the efficacy of an oncolytic herpes simplex virus 1 (HSV-1) in murine glioblastoma models,^17^ which may be reversed by transforming growth factor-β (TGF-β)^18^ and the proteasome inhibitor bortezomib.^19^ Although the role of NK cells in oncolytic adenovirus therapy has not been thoroughly explored, expression of Ad5 E1A protein can sensitize tumor cells to NK-mediated lysis,^20^ and several studies have implicated NK cells in the elimination of non-replicating E1-deleted adenovirus vectors.^21^^,^^22^

Here, we evaluated the role of NK cells in the efficacy of oncolytic adenoviral therapy in ovarian cancer. Species-specific replication of adenoviruses and the subsequent lack of *in vivo* immunocompetent model systems^23^ have hindered the understanding of immune responses to oncolytic adenoviral infections. To circumvent these shortcomings, we used primary NK cells isolated both from peripheral blood and ovarian cancer ascites to investigate the influence of NK cells in oncolytic adenoviral therapy in ovarian cancer. We show that NK cells augment the activity of oncolytic adenoviruses in a manner that is contact dependent and involves NK activating receptor DNAM-1 (DNAX accessory molecule-1). Furthermore, blockade of NK inhibitory receptor TIGIT also augments the effectiveness of oncolytic adenoviruses.

## Results

### Adenovirus Is Unable to Infect NK-92 and Primary Hematopoietic Cells from Ovarian Cancer Ascites

The ability of human adenoviruses to infect human immune cells, including NK cells, was assessed using Ad-GFP, a non-replicating adenovirus type 5 encoding green fluorescent protein (GFP) under the control of the CMV (cytomegalovirus) immediate early promoter, *dl*922-947 and NG-107, a GFP-encoding derivative of enadenotucirev. There was no detectable fluorescence in the established NK cell line NK-92 following Ad CMV GFP infection (Figure S1A), in contrast to a panel of ovarian cancer (OC) cells (p = 0.0002). Adenoviral protein expression was detected by immunofluorescence in TOV21G, but not peripheral blood NK (pNK) cells, after infection with *dl*922-947 (Figure S1B). Similarly, there was no detectable GFP fluorescence in CD45^+^ populations isolated from peripheral blood mononuclear cells (PBMCs) and ascites after incubation with Ad CMV GFP (Figure S1C), nor in pNK after incubation with NG-107 (Figure S1D). Thus, effects seen in subsequent experiments did not result from direct infection of immune cells by adenovirus.

### Upregulation of CD69, CD107a, and Interferon-γ (IFN-γ)

**Figure 1:**
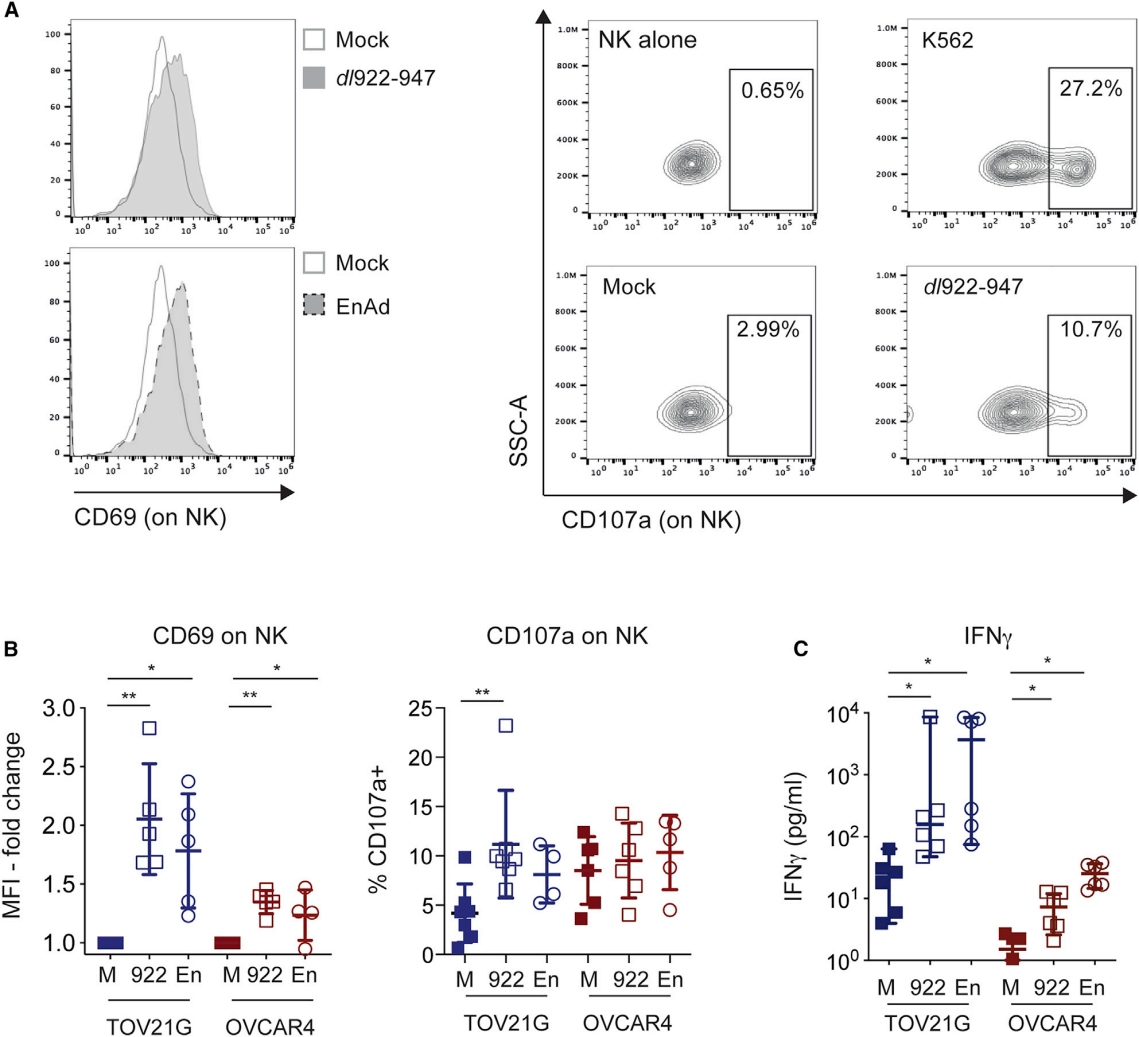
NK Cells Are Activated by Adenovirus-Infected OC Cells (A) Representative images of CD69 (left) and CD107a (right) expression on NK cells. (B) Summary analyses of independent experiments (n = 5) for CD69 (left) and CD107a (right) expression on NK cells (Wilcoxon signed rank tests). (C) Summary analyses of IFN-γ expression in pNK co-culture with OV-infected TOV21G (n = 6) and OVCAR4 (n = 2). Individual data points plotted for OVCAR4 (Wilcoxon signed rank test). M, mock; 922, *dl*922-947; En, enadenotucirev; K, positive control (co-culture with K562).

Because NK-92 cells have high basal expression of degranulation marker CD107a (Figure S2), the influence of oncolytic adenoviral infection on NK cell activation status was assessed in pNK cells. Co-culture of pNK with *dl*922-947-infected TOV21G and OVCAR4 cells led to upregulation of both CD69 and CD107a on pNK compared to mock-infected controls (Figure 1A). The CD69 average MFI (mean fluorescence intensity) increased significantly for both TOV21G (mean fold change 2.1, 95% confidence interval (CI) 1.4–2.7 p = 0.0022) and OVCAR4 (mean fold change 1.4, 95% CI 1.1–1.6, p = 0.0023). CD107a positivity also increased significantly from 4.2% to 11.2% for TOV21G (mean difference 7.0%, 95% CI 4.1%–9.9%, p = 0.001); a similar, albeit non-significant, trend was observed for OVCAR4 (Figure 1B). There were also significant increases in IFN-γ levels in cell-free supernatants following pNK co-culture (Figure 1C)—IFN-γ concentration increased 7-fold (median 22 pg/mL to 157 pg/mL, p = 0.03, n = 6 independent experiments) for TOV21G and nearly 4-fold (median 29 pg/mL to 114 pg/mL, p = 0.003) for OVCAR4 (Figure 1C).

Similarly, co-culture of pNK with enadenotucirev-infected TOV21G also led to a significant increase in CD69 expression (mean fold change 1.8, 95% CI 1.2–2.4, p = 0.0162) and IFN-γ release (median increase 3,679 pg/mL, p = 0.03) on pNK, as well as a trend toward CD107a upregulation (Figures 1B and 1C). Co-culture with enadenotucirev-infected OVCAR4 also led to increases in CD69 (Figure 1B, p = 0.034) and IFN-γ (Figure 1C, p < 0.0001) on pNK, although not CD107a. The observed NK activation for both viruses was dependent on contact with virus-infected OC cells, as exposure to conditioned medium alone did not upregulate CD69 or CD107a, nor did it increase IFN-γ production in any pNK group assessed (Figure S3).

### Oncolytic Adenovirus Augments Peripheral Blood and Ascites NK-Mediated Anti-tumoral Cytotoxicity

**Figure 2:**
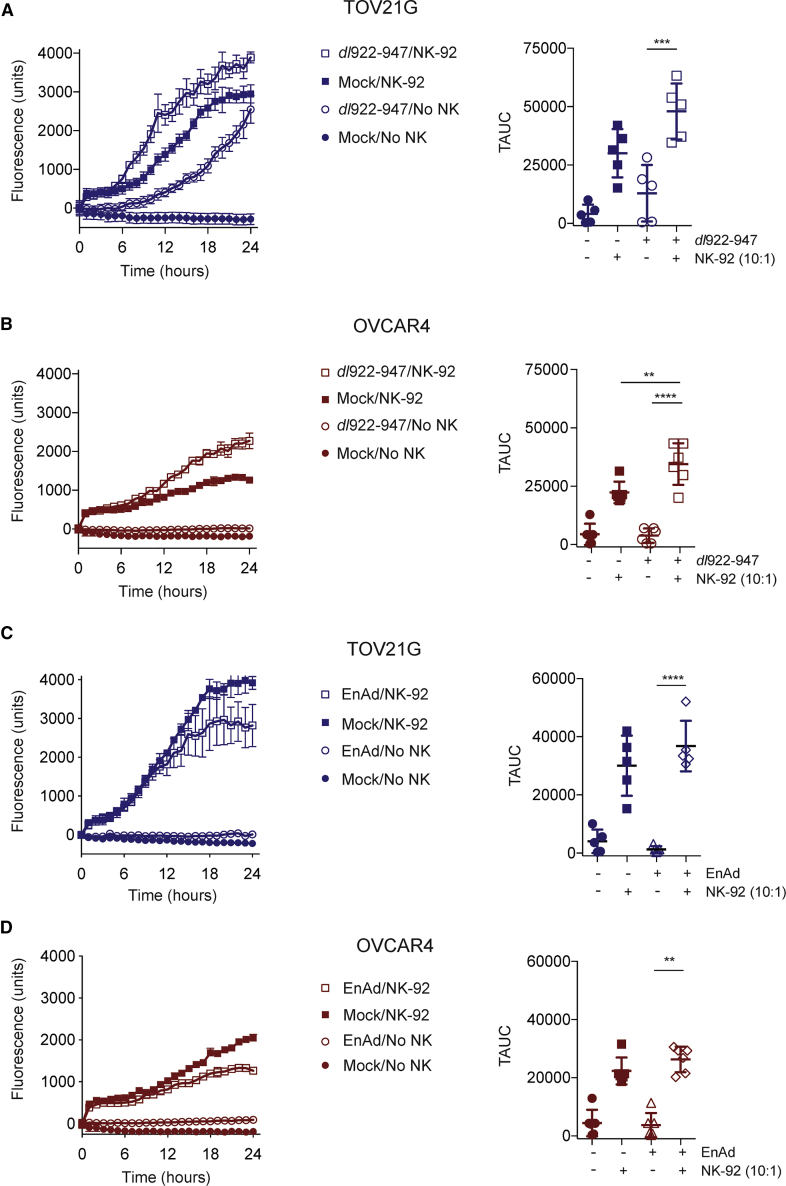
NK-92 Cells Increase Cytotoxicity of Oncolytic Adenovirus NK-92 cytotoxicity against TOV21G and OVCAR4 infected with *dl*922-947 (MOI 10, 48 h) or enadenotucirev (MOI 100, 48 h) was assessed by time-lapse microscopy. Fluorescent counts over time from individual experiments (left) and summary data (right) are presented: (A) TOV21G + *dl*922-947 (n = 5). (B) OVCAR4 + *dl*922-947 (n = 6). (C) TOV21G + enadenotucirev (n = 5). (D) OVCAR4 + enadenotucirev (n = 6). Summary data are expressed as total area under curve (TAUC) of fluorescent object counts over time (one-way ANOVA with multiple comparisons test).

We next used live cell imaging to quantify virus-induced NK cytotoxicity. In keeping with their high levels of basal activation (Figure S2), NK-92 alone led to significant killing of mock-infected TOV21G (Figure 2A) and OVCAR4 (Figure 2B) cells. However, NK-92 co-culture was able to augment both *dl*922-947 (increase in mean total area under curve [TAUC] of fluorescence over time 35,059 units, 95% CI 15,801–54,317, p = 0.001; Figure 2A) and enadenotucirev cytotoxicity (Figure 2C) in TOV21G cells compared to respective virus infection and NK alone. In OVCAR4 experiments, similar results were following both *dl*922-947 (increase in TAUC 30,632 units, 95% CI 21,405–39,860, p < 0.0001; Figure 2B) and enadenotucirev (Figure 2D) infection compared to virus and NK alone.

**Figure 3:**
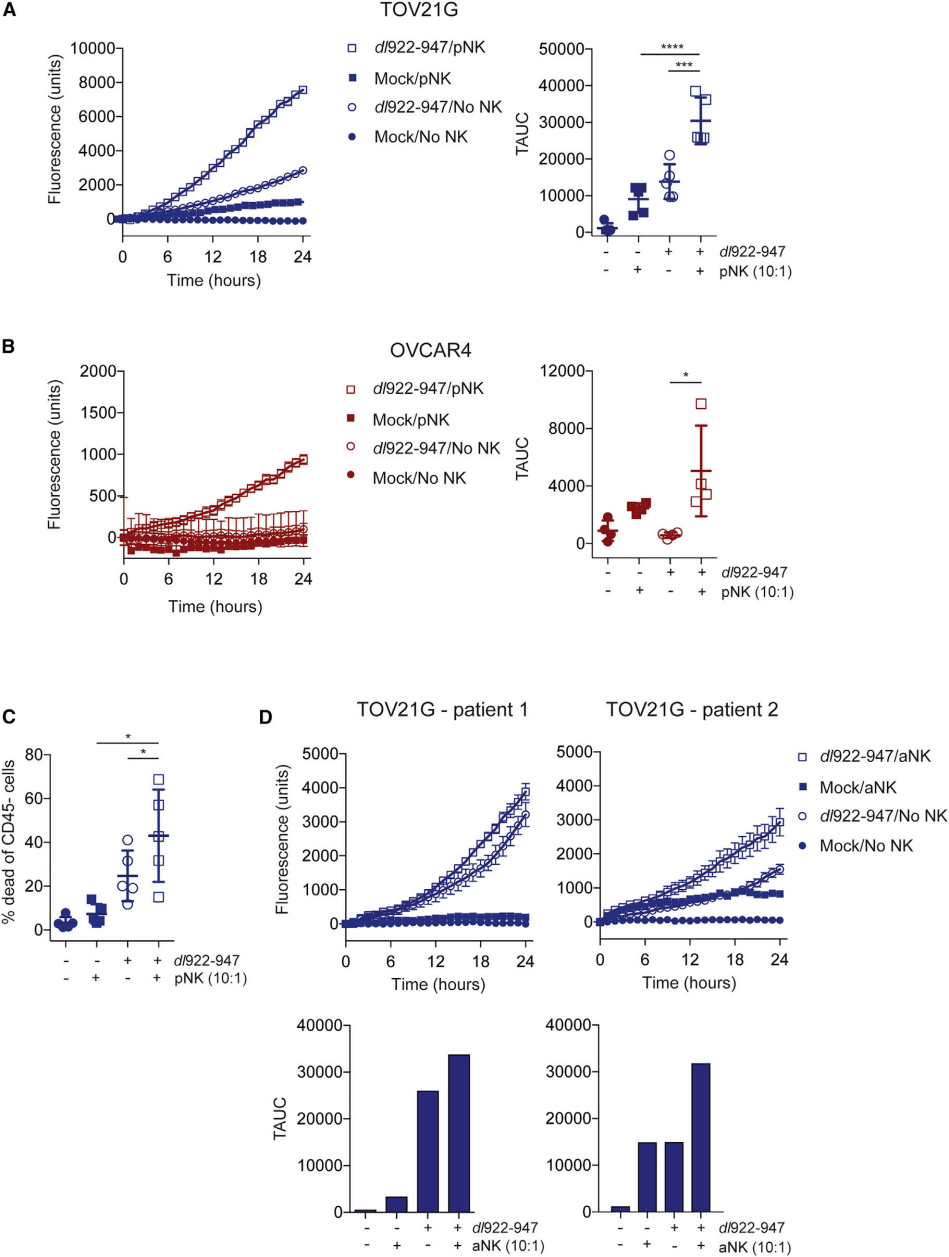
Peripheral Blood and Ascites NK Cells Increase the Cytotoxicity of Oncolytic Adenovirus Cytotoxicity of peripheral blood NK cells (pNK) or NK cells isolated from ovarian cancer ascites (aNK) against TOV21G and OVCAR4 infected with *dl*922-947 (MOI 10, 48 h) or enadenotucirev (MOI 100, 48 h) was assessed by time-lapse microscopy in (A), (B), and (D). Cytotoxicity of pNK against (A) TOV21G (n = 5) and (B) OVCAR4 (n = 4). Fluorescent counts over time from individual experiments (left) and summary data (right) are presented. (C) Cytotoxicity of pNK against TOV21G (n = 5) by flow cytometry. Data represent the percentage of dead CD45^−^ cells (TOV21G). (D) Cytotoxicity of aNK from patient 1 and patient 2 against TOV21G (one-way ANOVA with multiple comparisons test; error bars are not plotted when they are smaller than the symbol size).

Unlike NK-92 cells, pNK alone did not kill mock-infected ovarian cancer cells (Figures 3A and 3B). However, pNK were able to augment *dl*922-947 (Figure 3A) and enadenotucirev (Figure S4) cytotoxicity against TOV21G, when compared either to virus infection or pNK treatment alone. In OVCAR4 experiments, pNK also augmented *dl*922-947 (p = 0.016; Figure 3B), but not enadenotucirev (Figure S4), cytotoxicity compared with virus infection and NK alone.

To validate the live cell imaging results, we used flow cytometry to assess the viability of *dl*922-947-infected TOV21G cells. Significantly greater tumor cell death was observed when pNK were co-cultured with *dl*922-947-infected TOV21G, compared to virus infection or pNK treatment alone (Figure 3C; n = 5, mean difference = 18.3% and 35.7%, 95% CI 2.1%–34.6% and 7.2%–64.3%, p = 0.0342 and 0.024, respectively).

NK cells were also purified from ascites from two patients with advanced ovarian high-grade serous carcinoma (Figure S5) and co-cultured with TOV21G, with or without *dl*922-947 infection. As with pNK, purified ascites NK cells increased cytotoxicity of *dl*922-947-infected TOV21G, compared to *dl*922-947 infection and NK alone in two separate experiments (Figure 3D). Interestingly, ascites NK cells from patient 2 had a modest cytotoxic effect on uninfected TOV21G cells (Figure 3D).

### Oncolytic Adenoviruses Promote NK-Mediated Cytotoxicity of Uninfected Tumor Cells

**Figure 4:**
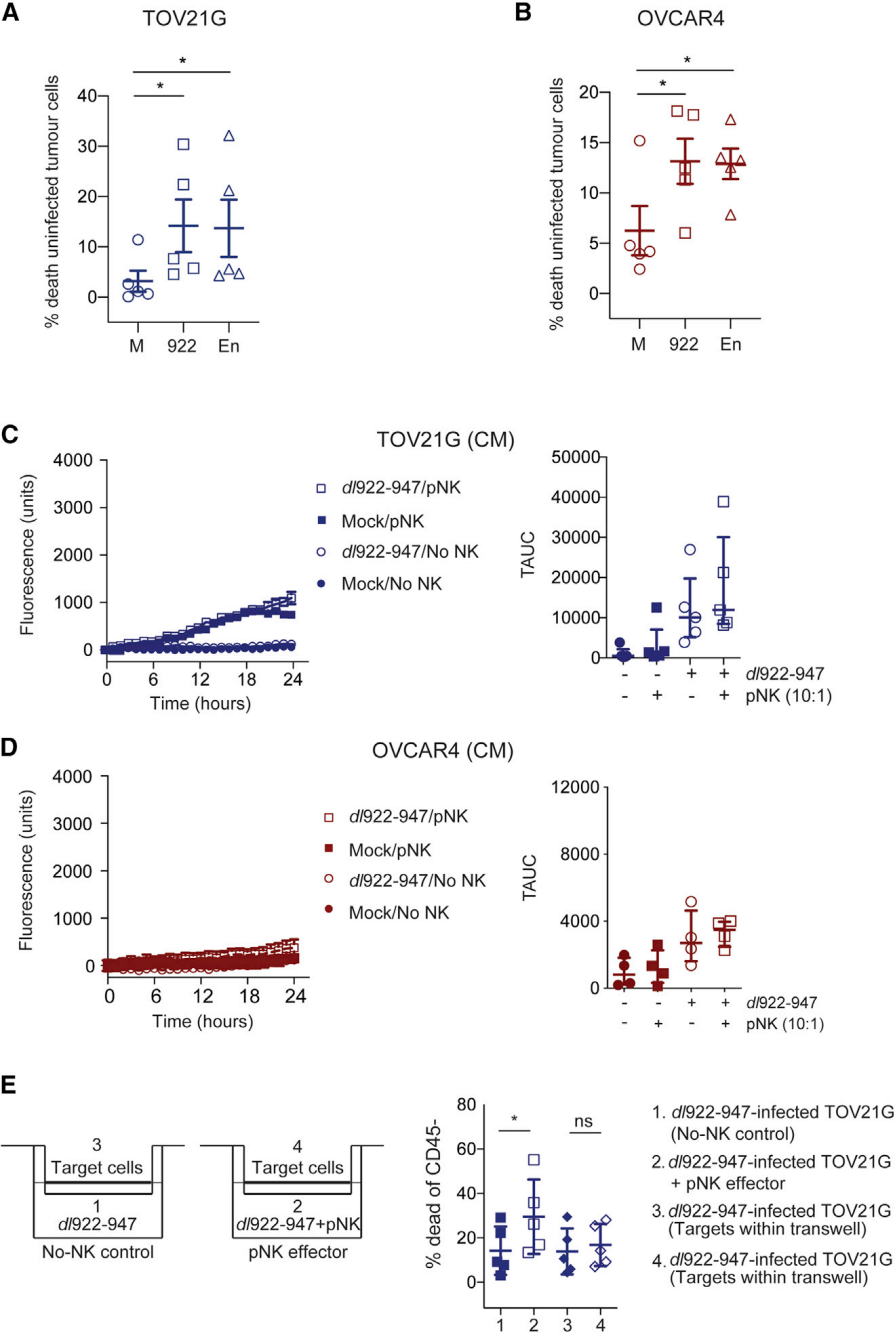
Activated NK Augmented Killing of Uninfected Target Cells and Contact Dependence of NK Cytotoxicity Cytotoxicity of activated peripheral blood NK cells (pNK) against cell-tracker marked and uninfected (A) TOV21G and (B) OVCAR4 was assessed by flow cytometry (n = 5 for each line; paired t tests). Cytotoxicity of pNK against uninfected (C) TOV21G (n = 5) and (D) OVCAR4 (n = 4) in the presence or absence of conditioned medium (CM) from *dl*922-947-infected TOV21G and OVCAR4, respectively. Cytotoxicity was assessed using live-cell imaging. Fluorescence counts over time from individual experiments (left) and summary data (right) are presented. (E) Target TOV21G were infected with *dl*922-947 (MOI 10, 48 h) in 12-well plates or in transwell inserts (group 1–4). Cell-free medium control (group 1) or pNK (group 2) were then incubated with virus-infected TOV21G for 18 h. Cell death of TOV21G in transwells (group 3 and 4), prevented from contact with NK cells but exposed to the environment of group 1 and 2, was compared by flow cytometry. The schematic representation of each group is shown on the left, with summary results (n = 5) on the right (paired t tests).

To evaluate whether NK cells activated by oncolytic adenoviruses could promote killing of uninfected tumor cells, we co-cultured uninfected tumor cells marked by cell tracker with pNK cells conditioned by exposure to either *dl*922-947- or enadenotucirev-infected tumor cells. Conditioned pNK cells induced additional cytotoxicity against both TOV21G (n = 5, p = 0.026 and 0.037, respectively) and OVCAR4 (n = 5, p = 0.030 and 0.028, respectively), when compared to pNK conditioned by mock-infected tumor cells (Figures 4A and 4B).

### Contact-Dependent Interactions between Oncolytic Adenovirus and NK

Soluble factors released from adenovirus-infected malignant cells alone were unable to augment NK cytotoxicity against uninfected target cells (Figures 4C and 4D), suggesting that direct contact between NK and virus-infected malignant cells was essential. To assess further whether cell-cell contact was a pre-requisite for the augmented NK anti-tumoral cytotoxicity, we compared cell death in *dl*922-947-infected cells in transwell inserts under different conditions (Figure 4E). Consistent with Figure 3, in the transwells in which NK cells and virus-infected TOV21G cells were in direct contact, there was significantly more cell death compared to virus infection alone (Figure 4E, group 1 versus 2; mean difference = 15.3%, 95% CI 6.8%–23.7%, p = 0.007). However, where there was no direct contact (Figure 4E, group 3 versus 4), there was no difference in the proportion of dead TOV21G cells within the transwell inserts, indicating a lack of contact-independent killing.

### Expression of Selected NK Ligands and Receptors after Oncolytic Adenovirus Infection

**Figure 5:**
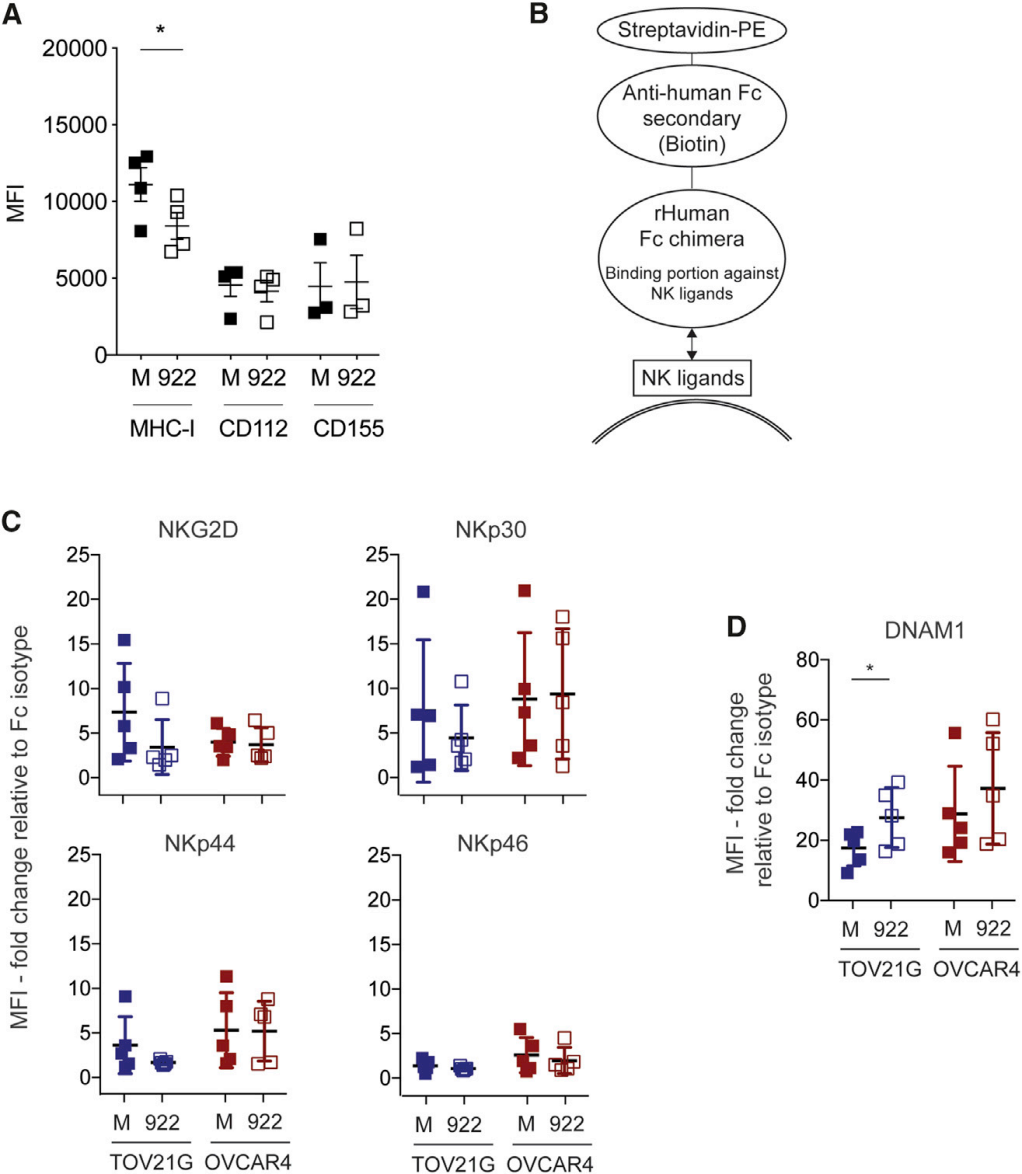
NK Ligand Expression following Ad5 Adenovirus Infection (A) TOV21G were infected with *dl*922-947 in triplicate (MOI 10, 48 h). Expression of evaluation of MHC class I (HLA-A, B, C; n = 4), CD112 (n = 4), and CD155 (n = 3) expression was assessed by flow cytometry (paired t test). Evaluation of NK ligand expression on TOV21G and OVCAR4 cells after *dl*922-947 infection (MOI 10, 48 h) using recombinant human Fc chimera proteins DNAM-1, NKG2D, NKp30, NKp44, and NKp46. (B) A schematic representation of the antigen-antibody complex. The recombinant human Fc chimera protein can bind to multiple ligands of the corresponding NK receptor. Summary data of independent experiments (n = 5 for each line) of (C) NKG2D, NKp30, NKp44, and NKp46 ligand expression, and (D) DNAM-1 ligand expression (paired t test).

MHC class I was downregulated on the surface of OC cells after infection with *dl*922-947 but not Ad3/11 enadenotucirev (Figure 5A; Figure S6A; p = 0.039) or non-replicating Ad5 adenovirus Ad LM-X (data not shown). We found no change in CD112, CD155 (Figure 5A), MICA/B, or CD58 (data not shown) expression following *dl*922-947 infection. In addition, using recombinant human Fc chimeras (Figure 5B), we did not detect any change in expression of ligands that bind to NKG2D, NKp30, NKp44, and NKp46 on TOV21G and OVCAR4 after infection with either *dl*922-947 or enadenotucirev (Figure 5C; Figure S6B).

**Figure 6:**
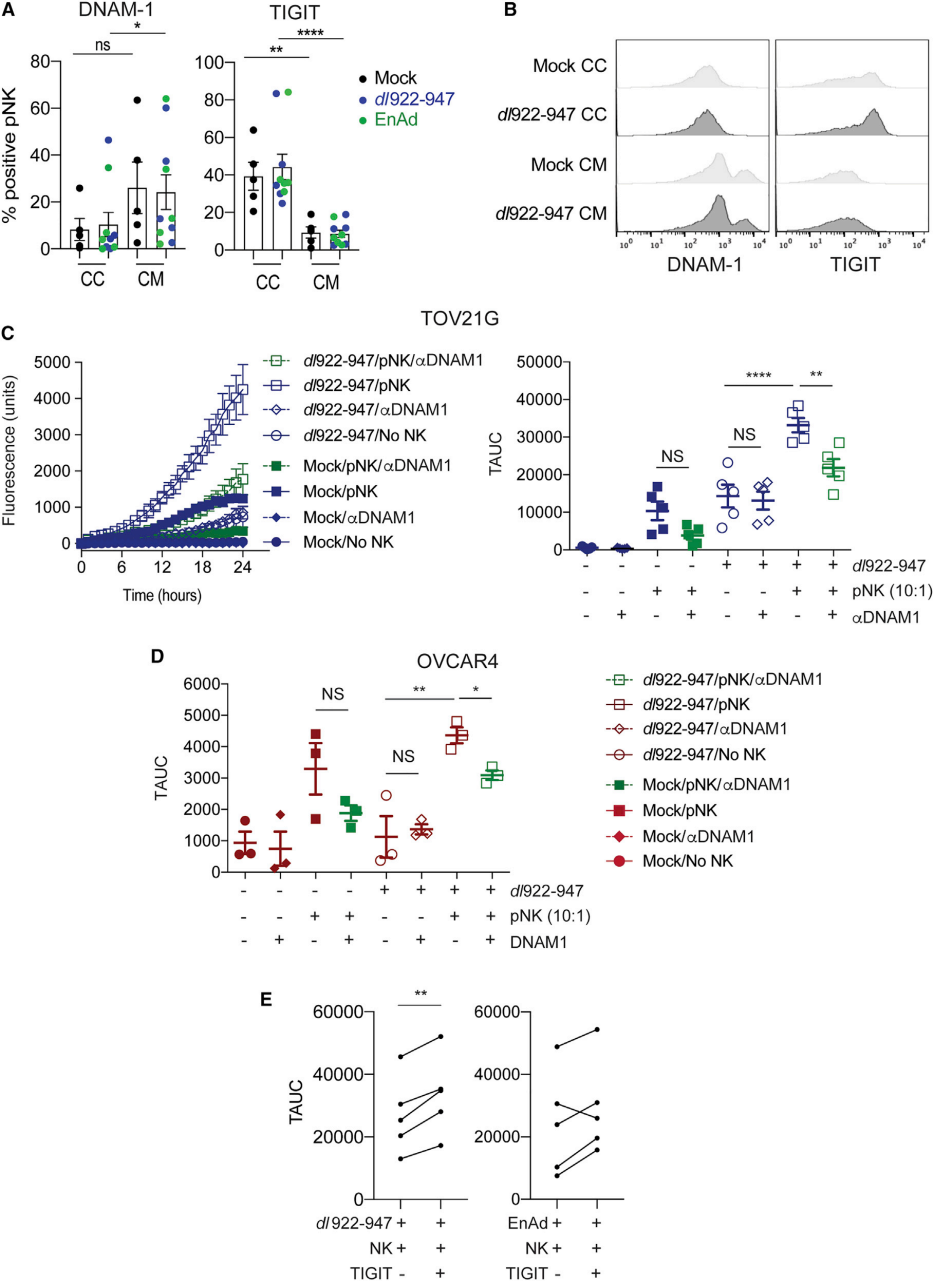
DNAM-1 Blockade Attenuates and TIGIT Blockade Augments NK-Mediated Cytotoxicity against Adenovirus-Infected Cells The changes of NK receptors on pNK after exposure to adenovirus-infected TOV21G or their conditioned media were evaluated by flow cytometry. (A) Summary analyses of independent experiments (n = 5) for DNAM-1 (left) and TIGIT (right) expression. (B) Representative images of DNAM-1 (left) and TIGIT (right) expression (Mann-Whitney U tests; CC, co-culture; CM, conditioned-medium). pNK were co-cultured with *dl*922-947-infected cells with or without a blocking anti-DNAM-1 antibody (10 μg/mL) and assessed by time-lapse microscopy. (C) Representative data of fluorescent object counts over time for TOV21G (left) and summary data (n = 5) of TAUC for TOV21G (right) and (D) OVCAR4 (n = 3) are shown (one-way ANOVA with multiple comparisons test). (E) pNK were co-cultured with OV-infected TOV21G cells with or without a blocking anti-TIGIT antibody (20 μg/mL) and assessed by time-lapse microscopy (n = 5, each line represents a unique donor; also see Figure S8).

Despite the absence of changes in CD112 and CD155 expression, we observed a significant increase in binding of DNAM-1 recombinant human Fc chimera on TOV21G following *dl*922-947 infection (Figure 5D; p = 0.024), but not enadenotucirev infection. A similar trend was observed in OVCAR4, although this did not reach statistical significance. To explore this further, we analyzed the expression of the paired receptors DNAM-1 and TIGIT, as well as CD96 and NKp46, on pNK cells following co-culture with TOV21G cells (both uninfected or adenovirus-infected) or conditioned medium (Figures 6A and 6B; Figure S7). There was no significant difference between the expression of NK receptors CD96 and NKp46 (Figure S7). However, co-culture of pNK with OC significantly reduced the expression of DNAM-1 (p = 0.04, Figures 6A and 6B) and enhanced the expression of TIGIT (p < 0.0001, Figures 6A and 6B) compared to conditioned-medium, independent of virus infection.

### The Role of DNAM-1 and TIGIT in NK Cytotoxicity against *dl*922-947-Infected Cells

Finally, we evaluated the effect of DNAM-1 and TIGIT blockade on NK cell activity against *dl*922-947-infected cells (Figures 6C and 6D). As previously, the addition of pNK augmented cytotoxicity of *dl*922-947 in both TOV21G and OVCAR4 cells. DNAM-1 blockade alone, in the absence of NK cells, had no effect on *dl*922-947 efficacy in either cell line. DNAM-1 blockade also had no significant effect on the efficacy of NK cells against uninfected cancer cells. In contrast, DNAM-1 blockade consistently reduced cytotoxicity in both *dl*922-947-infected TOV21G (decrease in TAUC 11,319 units; p = 0.008) and OVCAR4 (decrease in TAUC 1,271 units; p = 0.03) when co-cultured with NK cells. We also evaluated the effect of TIGIT blockade on NK cytotoxicity against OV-infected cells (Figure 6E), with mock-infected and NK-only groups as controls (Figure S8). Compared to NK alone, the addition of TIGIT blockade significantly augmented NK cytotoxicity against *dl*922-947-infected TOV21G (increase in TAUC 6,532 units, p = 0.003). A similar trend was observed for enadenotucirev-infected TOV21G (increase in TAUC 5,069 units), although the difference did not reach statistical significance.

## Discussion

Oncolytic viruses have the ability to induce potent immune responses, and recent data suggest that this could prime tumor cells for immune checkpoint blockade.^2^^,^^3^ However, NK cells have the ability to eliminate both virus-infected cells and malignant cells rapidly, which may also prevent the spread of OVs and reduce OV activity. It was previously shown that NK cells could impede anti-tumor activity of an oncolytic HSV vector *in vivo* via NKp30 and NKp46.^17^ In this study, we explored the role of NK cells in the activity of two different oncolytic adenoviruses, *dl*922-947 and enadenotucirev, using primary NK cells isolated from peripheral blood and ovarian cancer ascites—the use of primary cells was necessary given the species specificity of adenovirus replication.^23^

Overall, our results indicate that *dl*922-947 infection led to NK activation, as demonstrated by upregulation of CD69 and CD107a expression, as well as IFN-γ production. Enadenotucirev infection also promoted IFN-γ release and CD69 expression, with only modest changes in CD107a. For both viruses, this activation was contact-dependent, as exposure to cell-free conditioned medium from adenovirus-infected OC cells did not significantly increase CD69, CD107a, or IFN-γ.

Our results also indicate that all NK cells tested (NK-92, pNK, and ascites NK cells) augmented oncolytic adenovirus anti-tumor activities, compared to either NK cells or oncolytic adenovirus alone. This increase in cell death was observed both by conventional flow cytometry at a single time point and by time-lapse microscopy over 24 h. The changes were more pronounced for *dl*922-947 than enadenotucirev, consistent with the greater changes in NK activation markers observed. The effect was generally greater for pNK cells than was observed in the two ascites NK cells tested. It is possible that ovarian cancer ascites NK cells are less cytotoxic or have a more suppressed phenotype than pNK—previous evidence suggested that expression of activating receptors may be reduced on ascites NK cells in ovarian cancer, leading to impaired cytotoxicity.^24^ However, further experiments with a larger number of ascites samples would be required to understand more fully the activation status of ascites NK cells and their responsiveness to oncolytic virus infection. Like the NK cell activation, NK killing of oncolytic adenovirus-infected cells was contact-dependent—soluble factors released from virus-infected cells alone were insufficient to induce additional NK killing of uninfected target cells.

Infection by a wide variety of viruses can influence NK activity via inhibitory and activating NK receptors.^13^ Consistent with previous reports,^25^ we found that MHC class I molecules were downregulated after Ad5 oncolytic adenovirus infection, which could reduce anti-tumor immune responses. In contrast, MHC class I molecules were unchanged after Ad3/11 enadenotucirev and non-replicating Ad5 adenovirus infections. However, we saw no changes in MICA/B, which contrasts with previous data that suggested that the adenovirus protein E3 gp19K induced MICA/B downregulation in foreskin fibroblasts.^26^ In addition to the deletion in E1A CR2,^27^ *dl*922-947 also contains a 745 bp deletion in the E3B region that is replaced by 642 bp of heterologous DNA.^28^^,^^29^ However, the region encoding gp19K within E3A remains intact and the downregulation of MHC-I observed following *dl*922-947 infection suggests that gp19K is functional.^30^^,^^31^ The mechanism underlying differences in MHC class I expression after *dl*922-947 (Ad5) and enadenotucirev (Ad3/11) infection could also influence clinical anti-cancer efficacy and warrants further investigation.

We investigated the paired NK receptors DNAM-1 and TIGIT as DNAM-1 has previously been shown to play a key role in elimination of both malignant^32^^,^^33^ and CMV-infected cells^34^ by NK cells. In ovarian cancer specifically, the DNAM-1/CD155 interaction may be crucial for NK-mediated lysis.^35^ We found that DNAM-1 expression was decreased and TIGIT expression increased on NK cells following co-culture with ovarian cancer cells, independent of virus infection. However, the potential inhibitory effect of this change on NK was partially overcome by *dl*922-947 infection as DNAM-1 ligands were significantly upregulated after *dl*922-947 infection, as demonstrated by increased binding to a DNAM-1 Fc-chimera. Critically, we also found that DNAM-1 blockade significantly, albeit incompletely, reduced pNK cytotoxicity against *dl*922-947-infected malignant cells, but not against mock-infected cells.

Surprisingly, we were not able to demonstrate an increase in expression of the two main ligands for DNAM-1, CD155 (the Polio Virus Receptor/Nectin-like protein 5) and CD112 (Nectin-2), by flow cytometry following either *dl*922-947 or enadenotucirev infection. This suggests either the presence of additional DNAM-1 ligands that are upregulated following adenovirus infection or that adenovirus proteins may be able to bind to DNAM-1 causing NK cell activation. A preliminary assessment of the 36 adenovirus type 5 peptides identified three (III, IVa2, and V) with at least partial alignment with the consensus amino acid residues (DEGNYTC) critical for DNAM-1 interactions in Nectin/Nectin-like proteins, including CD112 and CD155,^36^ and thus potentially able to activate DNAM-1 (Figure S9). The interactions between viral and bacterial proteins and NK receptors have not been thoroughly investigated, and a systematic exploration of individual adenovirus proteins will be required to address these results fully. However, it has been shown that Fap2 protein derived from *Fusobacterium nucleatum* present in human colon can interact with TIGIT to inhibit NK cytotoxicity against colon cancer.^37^

We also found that TIGIT blockade augmented pNK cytotoxicity, reinforcing the importance of the DNAM-1/TIGIT axis in NK responses against cancer cells infected with oncolytic adenoviruses. TIGIT is an inhibitory NK receptor that competes with DNAM-1 and CD96 for ligand-binding.^38^^,^^39^ TIGIT is expressed on both T and NK cells, where its expression is associated with T cell exhaustion phenotypes.^38^ It is also upregulated in human malignancies and a number of anti-TIGIT antibodies (e.g., etigilimab/OMP-313M32, MTIG7192A, and AB154) are now being evaluated in early phase clinical trials as anti-cancer agents.^40^

In summary, oncolytic adenovirus-infected ovarian cancer cells were able to activate human NK cells and augment NK cytotoxicity *in vitro*. For *dl*922-947, an Ad5 oncolytic adenovirus, this augmented cytotoxicity was contact-dependent and involved modulating the interactions between activating NK receptor DNAM-1 and virus-infected malignant cells. Although enadenotucirev, an oncolytic group B adenovirus identified by its ability to propagate selectively in carcinoma cells and kill them rapidly,^41^ also augmented NK cytotoxicity, the effects were less marked than with *dl*922-947 infection and did not appear to be associated with DNAM-1. Further research will be required to evaluate additional NK receptor-ligand pathways involved in the augmented NK cytotoxicity observed, particularly for enadenotucirev. Our results highlight the lack of direct comparison of the efficacy of different oncolytic viruses and the importance of understanding the specific immune responses against each oncolytic virus for maximizing therapeutic benefits. Our demonstration that blockade of the paired NK inhibitory receptor TIGIT further augmented NK cytotoxicity against OV-infected cells suggests that the combination of oncolytic adenovirus and TIGIT blockade may be a viable treatment strategy in ovarian cancer.

## Materials and Methods

### Cell Lines and Tissue Culture

Ovarian cancer cell lines OVCAR4 (NCI, Frederick, MA), TOV21G (Fran Balkwill, Barts Cancer Institute, London, UK), erythroleukemia cell line K562 (Vignir Helgason, University of Glasgow, Glasgow, UK), and human NK cell line NK-92 (ATCC, Manassas, VA) were incubated at 37°C in 5% CO_2_. OVCAR4 and TOV21G were maintained in DMEM with 10% FBS, 2 mM L-Glutamine, and 100 μg/mL penicillin/streptomycin. NK-92 cells were maintained in MEM-alpha with 12.5% FBS, 12.5% horse serum, 2 mM L-Glutamine, and 5 ng/mL interleukin-2 (IL-2). K562 were maintained in RPMI with 10% FBS plus 2 mM L-Glutamine, and 100 μg/mL penicillin/streptomycin. All lines were tested regularly for mycoplasma infection. All human cancer cell lines were verified by short tandem repeat profiling at the Cancer Research UK Beatson Institute using the Promega GenePrint 10 system (Promega, Southampton, UK). Human NK cells were isolated, resuspended in RPMI with 10% FBS plus 2 mM L-Glutamine and 100 μg/mL penicillin/streptomycin, and used immediately without additional IL-2 or IL-15.

### Ethics Statement

Use of PBMCs isolated from samples from healthy blood donors was approved by the Scottish National Blood Transfusion Service (reference number 15-35). All donors gave written consent. Ascites samples from patients with ovarian cancer undergoing drainage for clinical purposes were collected under authority of the NHS Greater Glasgow and Clyde Biorepository (UK Health Research Authority Research Ethics Committee reference 10/S0704/60). Use of ascites samples for this project was then authorized by the NHS Greater Glasgow and Clyde Biorepository Access Committee (reference 16/WS/0207). All patients gave written consent and samples were anonymized.

### Isolation of Peripheral Blood and Ascites-Derived NK Cells

pNK cells were isolated from PBMCs using EasySep Human NK Cell Enrichment Kits (19055; StemCell Technologies, Canada) according to the manufacturer’s instructions. Human ovarian cancer ascites samples were centrifuged at 2,500 rpm for 15 min at 18°C (JS-4.2, Beckman-Coulter, USA) in 250 mL centrifuge tubes. The cell pellet was enriched using EasySep Human NK Cell Enrichment Kits before fluorescence-activated cell sorting (FACS) based on extracellular cell surface markers of NK cells (CD45^+^CD3^−^CD56^+^). The purity of primary NK cells (>90%) was confirmed by flow cytometry.

### Adenoviruses

The E1A CR2-deleted Ad5 vector *dl*922-947 and enadenotucirev, an Ad3/Ad11p chimeric virus generated by directed evolution, have both been previously described.^11^^,^^29^ Enadenotucirev was provided by PsiOxus Therapeutics (Oxford, UK). Ad CMV GFP is an E1-deleted replication-deficient adenovirus type 5 vector expressing eGFP under the CMV immediate/early promoter. Reporter enadenotucirev-based virus NG-107 (SSA promoter) also encodes eGFP and was generated by Marino et al.^42^ Ad LM-X, a replication-defective adenovirus type 5 has been described previously.^43^ Viruses were expanded in 293 cells before cesium chloride (CsCl) purification, as previously described.^10^^,^^11^^,^^29^^,^^43^ Viruses were then quantified by measuring particle counts and tissue culture inhibitory dose 50% (TCID50) assays. In all infection experiments, multiplicity of infection (MOI) was defined as plaque-forming units (pfu)/cell. Cells were infected for 48 h prior to co-culture with NK cells for a further 24 h. MOIs were selected based upon dose response curves with the chosen cell lines as determined by MTT (3-(4,5-dimethylthiazol-2-yl)-2,5-diphenyltetrazolium bromide) assays post-infection (Figures S10 and S11), as previously described.^10^

### Infectability of NK Cells

NK-92, PBMCs, cells from ascites, purified pNKs, and OC lines (including TOV21G) were incubated with Ad CMV GFP (MOI 10), *dl*922-947 (MOI 10), and NG-107 (MOI 100) for 48 h before flow cytometric and immunofluorescence assessment of infectability. Because there is not a GFP-encoding derivative of *dl*922-947, adenovirus protein expression was assessed by immunofluorescence using an anti-adenovirus primary antibody (Ab36851; 1 in 100; Abcam, UK). Cells were counterstained with DAPI. Imaris software (Version 9.3.1; Bitplaine, Switzerland) was used to identify immunofluorescent cells. Briefly, individual cells were identified by DAPI (4′,6-diamidino-2-phenylindole) staining and counted. For each experiment, the fluorescence intensity (GFP or Alexa Fluor 594) of mock-infected pNK cells was used to define the minimum fluorescence intensity of positive (i.e., infected) cells. The same algorithm was then applied to all images in the same experiment and positive/infected cells were identified and counted. To allow direct comparisons between groups and replicates, we calculated percentages of positive/infected cells.

### Evaluation of CD107a, CD69, and NK Receptors

For co-culture experiments, TOV21G and OVCAR4, with or without *dl*922-947 (MOI 10, 48 h) and enadenotucirev (MOI 100, 48 h) infection, were incubated with 5 × 10^5^ PBMCs from healthy donors in 96-well plates at 1:1 ratio in a total volume of 100 μL. Similarly, for the conditioned medium experiments, cell-free conditioned medium from *dl*922-947 (MOI 10, 48 h) and enadenotucirev (MOI 100, 48 h) infected TOV21G and OVCAR4 was incubated with 5 × 10^5^ PBMCs from healthy donors in 96-well plates in a total volume of 100 μL.

To collect cell-free conditioned medium, we plated 10^6^ OC cells (OVCAR4, TOV21G) on 6 cm plates before infection with *dl*922-947 (MOI 10) or enadenotucirev (MOI 100) in a total volume of 1.5 mL. Cell-free medium was collected 48 h after infection. For CD107a and CD69, the mixtures were incubated for 5 h at 37°C in the presence of monensin (GolgiStop, 1:1,000, cat number: 554724, BD Biosciences, Berkshire, UK) and conjugated antibodies for CD3, CD56, and degranulation marker CD107a or activation marker CD69 in a total volume of 150 μL. Cells were washed and stained by fixable viability dye before fixation and flow cytometric evaluation. For cell surface NK receptors NKp46 (clone 9E2), CD96 (clone NK92.39), DNAM-1 (clone 11A8), and TIGIT (clone A15153G), the mixtures were incubated for 18 h at 37°C before flow cytometric evaluation.

### IFN-γ Detection by ELISA

In co-culture experiments (CC), 10^4^ target cells (TOV21G and OVCAR4 infected with *dl*922-947 [MOI 10] or enadenotucirev [MOI 100] for 48 h) were incubated with 10^5^ pNK cells or no-NK culture medium (RPMI with 10% FBS) for 24 h in a 96-well plate at a total volume of 200 μL (E:T ratio 10:1). In the conditioned medium experiments (CM), cell-free conditioned medium from mock-infected or virus-infected cells was added to 10^5^ pNK or no-NK culture medium (RPMI with 10% FBS) for 24 h in a 96-well plate at a total volume of 200 μL. At the end of the incubation period (24 h) for both groups (CC and CM), the plate was centrifuged (430 g, 5 min), and the supernatant collected for IFN-γ assessments by ELISA (Human IFN-γ ELISA MAX Deluxe kit, cat number 430104; Biolegend, San Diego, CA), according to the manufacturer’s instructions.

### Assessment of NK Cytotoxicity by Live Cell Analysis System

NK-92, pNK, or ascites NK cells were co-cultured with *dl*922-947-infected (MOI 10, 48 h post-infection), enadenotucirev-infected (MOI 100, 48 h post-infection), mock-infected TOV21G and OVCAR4 or conditioned medium from each group at an effector-to-target (ET) ratio of 10:1 in flat-bottom 96-well plates in triplicate at 3,000 cells per well. Fluorescent Cytotox nucleic acid dye was used for identification of dead cells. Images were assessed using an IncuCyte Live Cell Analysis System (Essen Biosciences, Ann Arbor, MI) to identify the dead cells (as fluorescent objects) in each image (two 10× magnification images from different areas per well were captured every hour). Dead NK cells were excluded from analyses based on size (the minimal surface area for a fluorescent object to be included was 125 μm^2^). The TAUC of fluorescent object counts over time was used to quantify overall NK killing. Target-only and NK-only controls were routinely used for these experiments.

### Assessing NK Cytotoxicity by Flow Cytometry

TOV21G were plated in 24-well plates at 10^4^ cells per well. After overnight incubation, cells were either mock-infected or infected with *dl*922-947 (MOI 10). 48 h post-infection, NK-92 or human pNK were added (ET ratio of 10:1). Cells were harvested after 18-h co-culture, washed once and stained with surface marker CD45 for NK cells (Clone 2D1, Biolegend, San Diego, CA) and fixable viability dye (cat number 423113, Biolegend, San Diego, CA). Cell death in CD45-negative target TOV21G cells was determined by flow cytometry. Target-only and NK-only controls were also used in these experiments.

### NK-Mediated Killing of Uninfected Tumor Cells

Target TOV21G and OVCAR4 cells (2 × 10^4^/well) were plated on 12-well plates. After mock, *dl*922-947 (MOI 10, 48 h) or enadenotucirev (MOI 100, 48 h) infection, pNK (ET ratio 5:1) or no-NK culture medium (negative control) was added to each well. Uninfected cells (TOV21G and OVCAR4, 2 × 10^4^/well) were marked by CellTracker Green CMFDA Dye (2 μM, C7025, Thermo Fisher Scientific, USA) and added to target cells 4 h after initiation of NK/infected target cell co-culture. After a further 18-h incubation, supernatants and cells were collected and centrifuged (430 g for 5 min). Viability of uninfected CellTracker green cells was determined by flow cytometry. Additional NK-mediated killing was calculated by subtracting the percentage of cell death in the no-NK controls from that in the pNK groups.

### Transwell Assays to Assess NK Bystander Killing

Contact-dependence of pNK cytotoxicity against *dl*922-947-infected TOV21G was evaluated by assessing cell death of *dl*922-947-infected TOV21G in 0.4 μm transwells. TOV21G (10^4^/well) were plated on the bottom of a 12-well plate and on the 0.4-μm-pore polycarbonate 12-well transwell inserts (12 mm membrane diameter; cat number: 3401, Corning, USA). After mock or *dl*922-947 infection (MOI 10, 48 h), pNK (10^5^; ET 10:1), or no-NK culture medium (negative control) was added to the bottom of the well. After an 18-h incubation, the inserts were carefully removed without spillage into a separate 12-well plate. The supernatants and trypsinized TOV21G of each group were combined and centrifuged (430 g for 5 min) before staining with viability dye and CD45 antibody (Clone HI30; Biolegend, San Diego, CA). Cell death in CD45-negative target TOV21G cells was determined by flow cytometry.

### Recombinant Human Fc Chimera Protein and Flow Cytometry for Detection of NK Ligands

As multiple NK ligands can bind to a single NK receptor, NK ligand expression on infected OC cells was evaluated using recombinant human Fc chimera proteins. Recombinant human Fc chimera proteins consist of human immunoglobulin G_1_ (IgG_1_) and the relevant binding portion of the proteins of interest. These binding portions can bind with multiple relevant NK ligands of the corresponding NK receptors, e.g., DNAM-1. As the affinity of these chimeric proteins and target ligands could be influenced by treatment conditions,^44^^,^^45^ the fold change in MFI between relevant Fc chimera and control Fc protein was used to compare differences between groups.

Cells were stained with recombinant human Fc chimera proteins (R&D Systems, Minneapolis) or conjugated antibodies (all from Biolegend, San Diego, CA) against MHC class I proteins (clone W6/32), CD112 (clone TX31), and CD155 (clone SKII.4). As the antibody against human Fc-protein was conjugated with biotin, tertiary antibody staining was assessed with Streptavidin-PE in 50 μL at 1:1,000 for 30 min at 4°C–8°C in the dark (Figure 5B, cat number 405203, Biolegend, San Diego, CA). Cells were washed twice with 150 μL of FACS buffer, centrifuged (430 g, 5 min, 4°C) and supernatant discarded.

### DNAM-1 and TIGIT Blockade

Human NK cells were rested in culture medium for 1 h, with or without anti-DNAM-1 (MAB666, R&D Systems, Minneapolis, MN; final concentration 10 μg/mL)^17^ or anti-TIGIT (MAB78981, R&D Systems, Minneapolis, MN; final concentration 20 μg/mL) antibody, before co-culture with target cells (with or without OV infections). Cell-free medium with appropriate antagonists and no target cell controls was routinely used as control.

### Statistical Analyses

All statistical analyses were performed using Prism (v8.0, GraphPad, La Jolla, CA). Data are presented as mean ± SEM unless otherwise stated. Paired analyses (one-way ANOVA with multiple comparison tests, t tests, or their non-parametric equivalent) were used for analysis of repeated experiments, and p < 0.05 was considered significant throughout. Unless otherwise stated, each symbol/point represents the average value of an independent experiment. In all figures, ns = not significant, ∗p < 0.05, ∗∗p < 0.01, ∗∗∗p < 0.001, ∗∗∗∗p < 0.0001.

## Author Contributions

Conceptualization, E.Y.L.L., I.A.M. Data curation, E.Y.L.L., I.A.M., D.P.E., S.D., M.F., P.S., J.N., S.M. Formal analysis, E.Y.L.L., I.A.M. Funding acquisition, E.Y.L.L., I.A.M., G.G., D.M.D. Methodology, E.Y.L.L., P.R.K., C.H., L.M.C., D.M.D., G.G. Project administration, E.Y.L.L., I.A.M. Resources, C.H., K.F., D.P.E., S.D., M.F., P.S. Supervision, I.A.M., D.M.D., G.G. Visualization, E.Y.L.L., I.A.M. Writing – original draft, E.Y.L.L., I.A.M. Writing – review & editing, all authors.

## Conflicts of Interest

K.F. is an employee of PsiOxus Therapeutics Limited. The remaining authors declare no competing interests.

## Supplemental Information

Document S1. Figures S1–S11

Document S2. Article plus Supplemental Information
