# Supplementary material for: NK Cells Augment Oncolytic Adenovirus Cytotoxicity in Ovarian Cancer
Source: Mol Ther Oncolytics. 2020 Feb 15;16:289–301. doi: 10.1016/j.omto.2020.02.001 (PMC7068056; doi:10.1016/j.omto.2020.02.001)

OMTO, Volume *16*

Supplemental Information

NK Cells Augment Oncolytic Adenovirus Cytotoxicity in Ovarian Cancer

Elaine Y.L. Leung, Darren P. Ennis, Philippa R. Kennedy, Christopher Hansell, Suzanne Dowson, Malcolm Farquharson, Pavlina Spiliopoulou, Jaya Nautiyal, Sophie McNamara, Leo M. Carlin, Kerry Fisher, Daniel M. Davis, Gerard Graham, and Iain A. McNeish

Supplementary Figure Legends

Figure S1. NK cells and other CD45+ cells cannot be infected with Ad5 vectors

1A. NK-92 and a panel of ovarian cancer cell lines were infected with non-replicating Ad CMV GFP vector (MOI 10). Green fluorescence was assessed by flow cytometry 24h post-infection (paired t-test).
1B. Peripheral blood NK (pNK) and TOV21G were infected with *dl*922-947 (MOI 10) for 48 hours before staining with an anti-adenovirus antibody (Alexa Fluor 594 secondary antibody) and assessed by quantitative immunofluorescence. Left: summary data (paired t test: *;$p<0.05$), Right: representative images.
1C. PBMC and CD45^{+} cells from ovarian cancer ascites were infected with Ad CMV GFP vector (MOI 10) for 24 hours. Green fluorescence was assessed by flow cytometry, with TOV21G as positive control (n=2).
1D. Peripheral blood NK (pNK) and TOV21G were infected with NG-107 (MOI 100, GFP-expressing derivative of enadenotucirev) for 48 hours and imaged using quantitative immunofluorescence Left: summary data (paired t test: *;$p<0.05$), Right: representative images.

Figure S2. Flow cytometric assessment of baseline CD107a expression in NK-92.

NK-92 cells were incubated with monensin (1:1000), with or without phorbol myristate acetate (PMA), for 5 hours at 37 ^{0}C before evaluation for degranulation marker CD107a. Isotype of CD107a was used as a negative control.

Figure S3. NK cell activation requires cell-cell contact

Peripheral blood NK (pNK) cells were incubated for 6 hours with conditioned medium harvested from TOV21G and OVCAR4 cells infected with *dl*922-947 (922 – MOI 10, 48h) or mock infection (M). Expression of CD69 (left) or CD107a (middle) was assessed by flow cytometry. In addition, IFN□ release was assessed by ELISA following incubation of pNK cells with cell-free conditioned medium from TOV21G and OVCAR4 cells that had been *dl*922-947 (922 – MOI 10, 48h), enadenotucirev (En, MOI 100, 48h) or mock infection (M) (right).

Figure S4. pNK cells and enadenotucirev-induced cytotoxicity

TOV21G (left) and OVCAR4 (right) cells were infected with enadenotucirev (EnAd, MOI 100 48h), followed by co-culture with pNK cells (ET ratio 10:1). Cells were imaged over 24 hours as detailed in Materials and Methods. Cell viability was calculated as total area under curve (TAUC) for fluorescent green objects (paired t test).

Figure S5. Flow cytometry gating strategy for isolation of ascites NK cells after a two-step enrichment process.

The gating strategy for cell-sorting for isolation and enrichment of ascites NK cells. The proportions of CD45+CD3-CD56+ before any cell sorting (top row), after MACS (middle row) and after MACS and purified with FACS (bottom row) are illustrated.

Figure S6. NK ligand expression following enadenotucirev infection

A) TOV21G were infected with enadenotucirev in triplicate (MOI 100, 48h). Expression of evaluation of MHC Class I (HLA-A, B, C; n=4), CD112 (n=4) and CD155 (n=3) expression was assessed by flow cytometry.

Evaluation of NK ligand expression on TOV21G and OVCAR4 cells after enadenotucirev (MOI 100, 48h) infection using recombinant human Fc chimera proteins DNAM-1, NKG2D, NKp30, NKp44 and NKp46. B) the summary data of independent experiments (right, n=5) are shown.

Figure S7. Co-culture with virus-infected OC changes the expression of NK receptors assessed.

Peripheral blood NK (pNK) cells were co-cultured (CC) with virus-infected TOV12G and OVCAR4 or incubated with conditioned medium (CM) harvested from TOV21G and OVCAR4 cells infected with adenoviruses for 18 hours. Expression of NK receptors DNAM-1, TIGIT, CD96 and NKp46. 922 = *dl*922-947, EnAd = enadenotucirev, Ad5 WT = Wild-type Ad5.

Figure S8. TIGIT-blockade augments NK-mediated cytotoxicity against adenovirus-infected cells

pNK were co-cultured with OV-infected cells with or without a blocking anti-TIGIT antibody (20μg/mL) and assessed by time-lapse microscopy. No NK, mock-infected and NK-only groups were used as controls (n=5). 922 = *dl*922-947, EnAd = enadenotucirev (One-way ANOVA with multiple comparisons test).

Figure S9. BLAST alignment

The critical amino acids for interacting with DNAM-1 from CD112 (Nectin-2 residues 134 - 140) and CD155 (Nectin-like 5 residues 117 – 123) {Liu, 2012 #7163} were aligned against all 36 expressed proteins from human adenovirus-type 5. Only proteins III (reference AP_000206.1), IVa2 (reference AP_000201.1) and V (reference AP_000208.1) yielded any alignment.

Figure S10. Dose response curves of *dl*922-947 infection

OVCAR4 and TOV21G cells were infected in triplicate with *dl*922-947 (MOI 0.001 – 1000 pfu/cell). Cell survival was assessed 72 hours post-infection by MTT assay.

Figure S11. Enadenotucirev cytotoxicity in ovarian cancer cell lines

OVCAR4 (n=4), TOV21G (n=6) and primary ovarian high grade serous carcinoma lines (n=11) were infected in triplicate with enadenotucirev (MOI 0.1 – 1000 pfu/cell). Cell survival was assessed 72 hours post-infection by MTT assay. Each data points represents IC50 in pfu/cell from one experiment.

Supplementary Figures

S1A

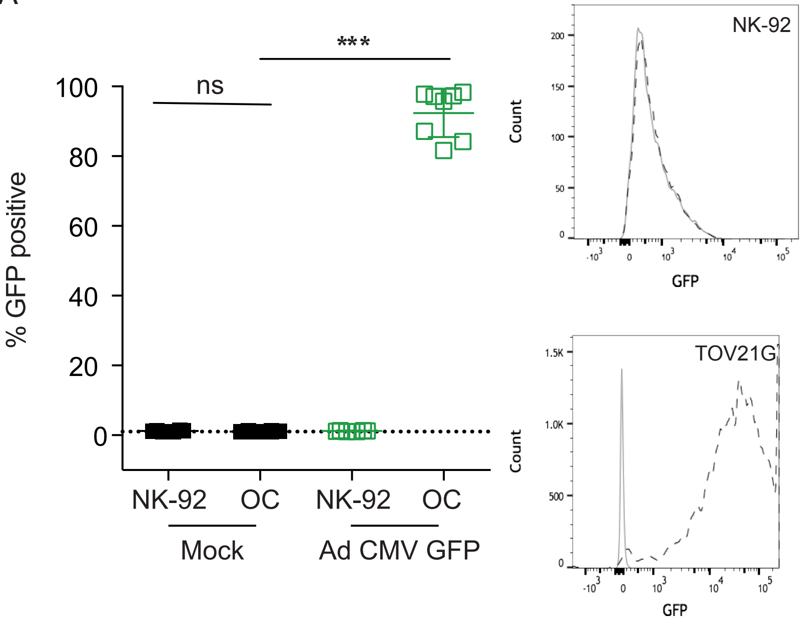

S1B

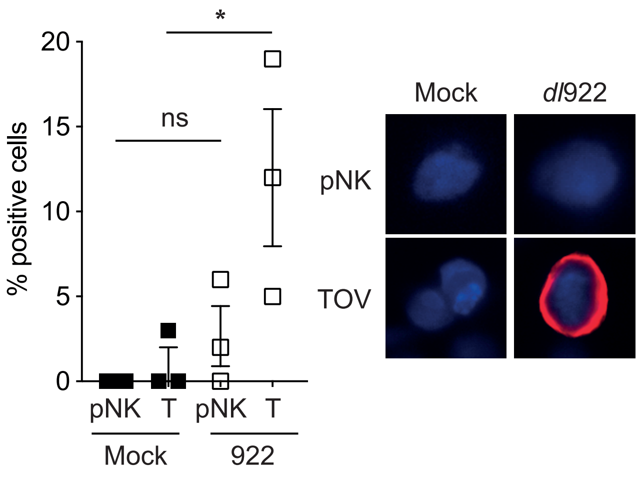

S1C

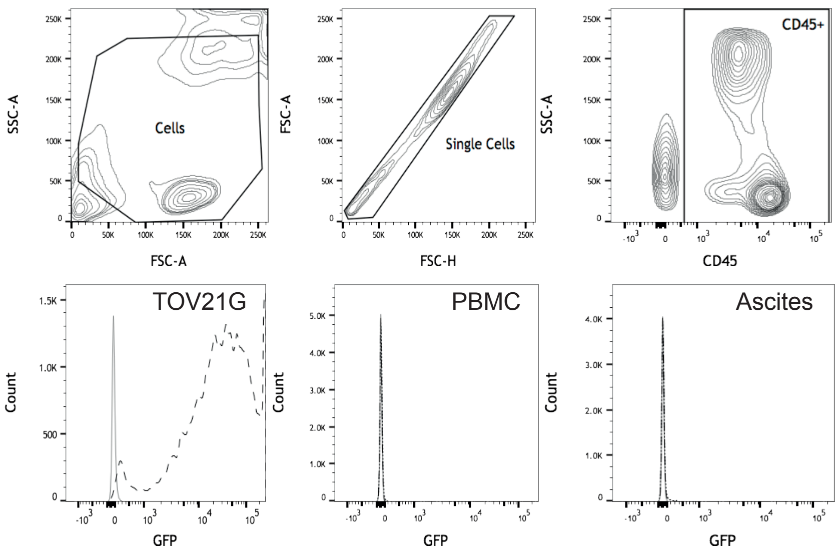

S1D

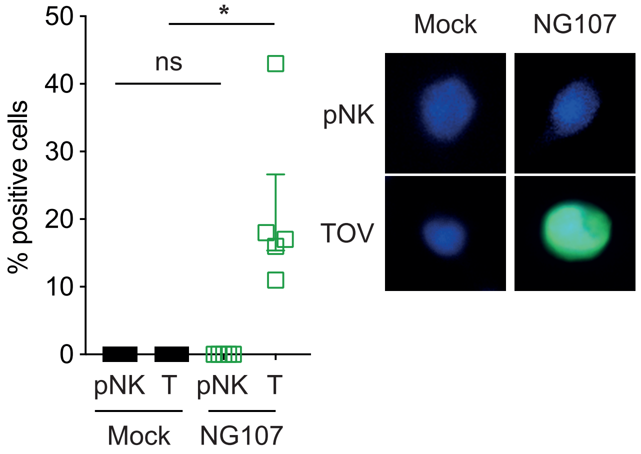

S2

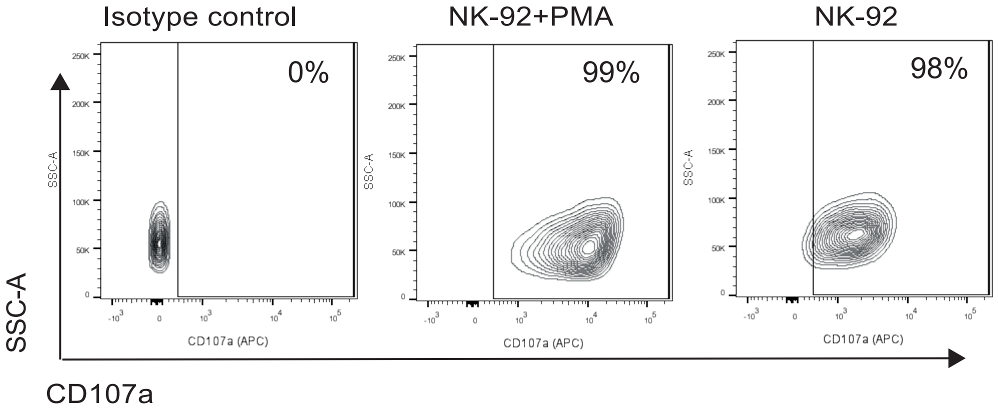

Supplementary Figures

S3

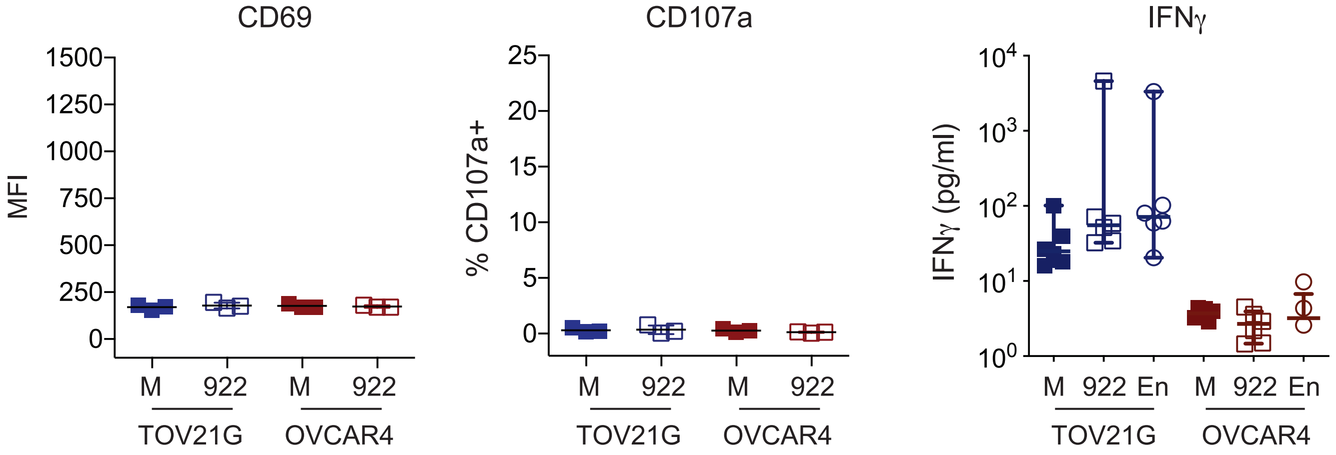

S4

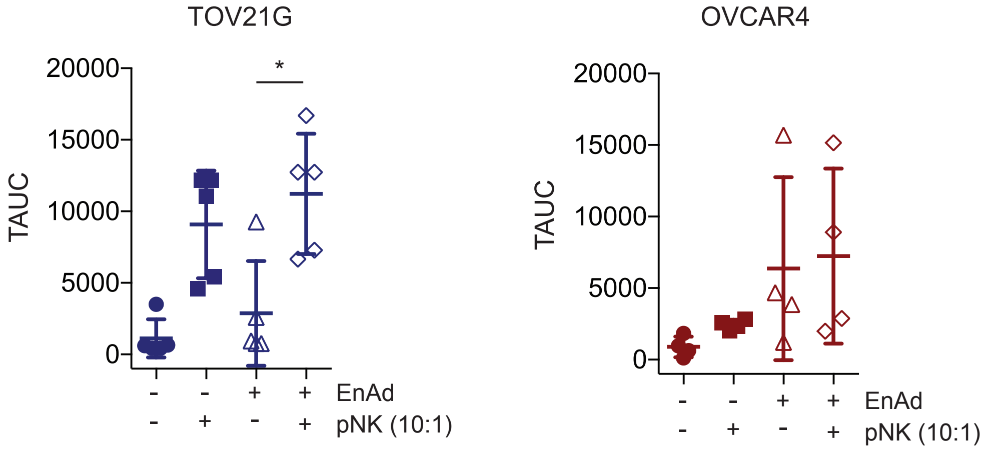

Supplementary Figures

S5

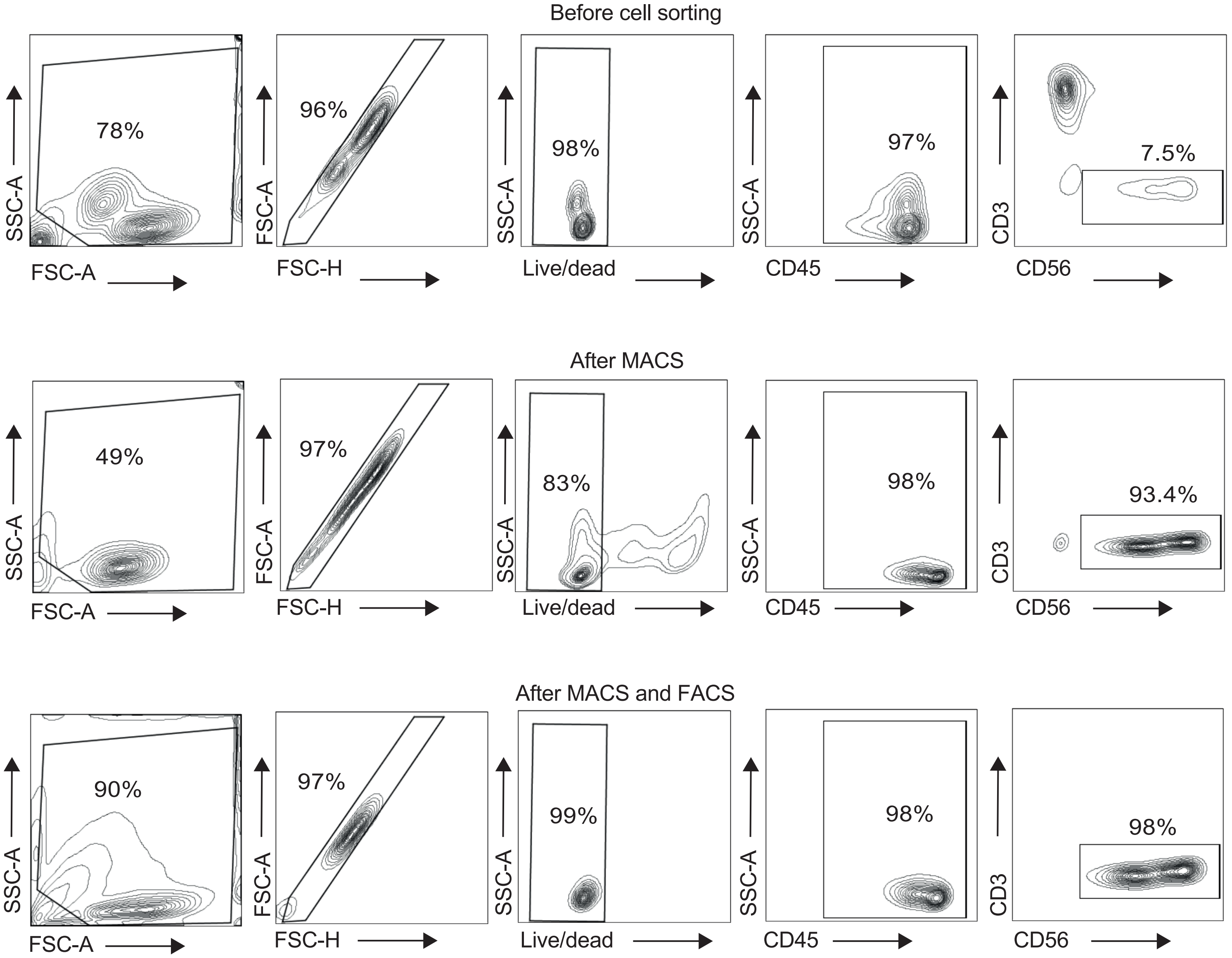

S6

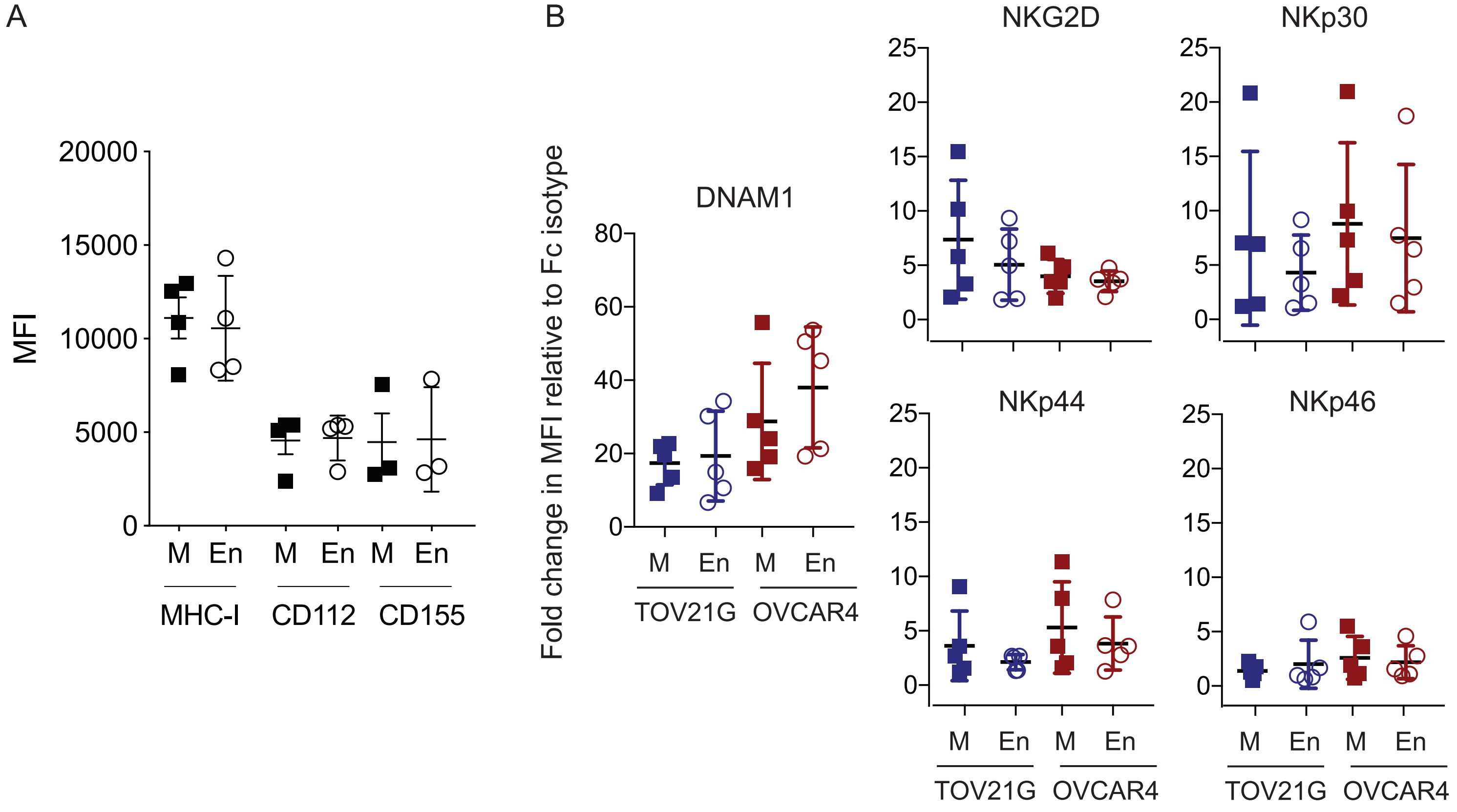

Supplementary Figures

S7

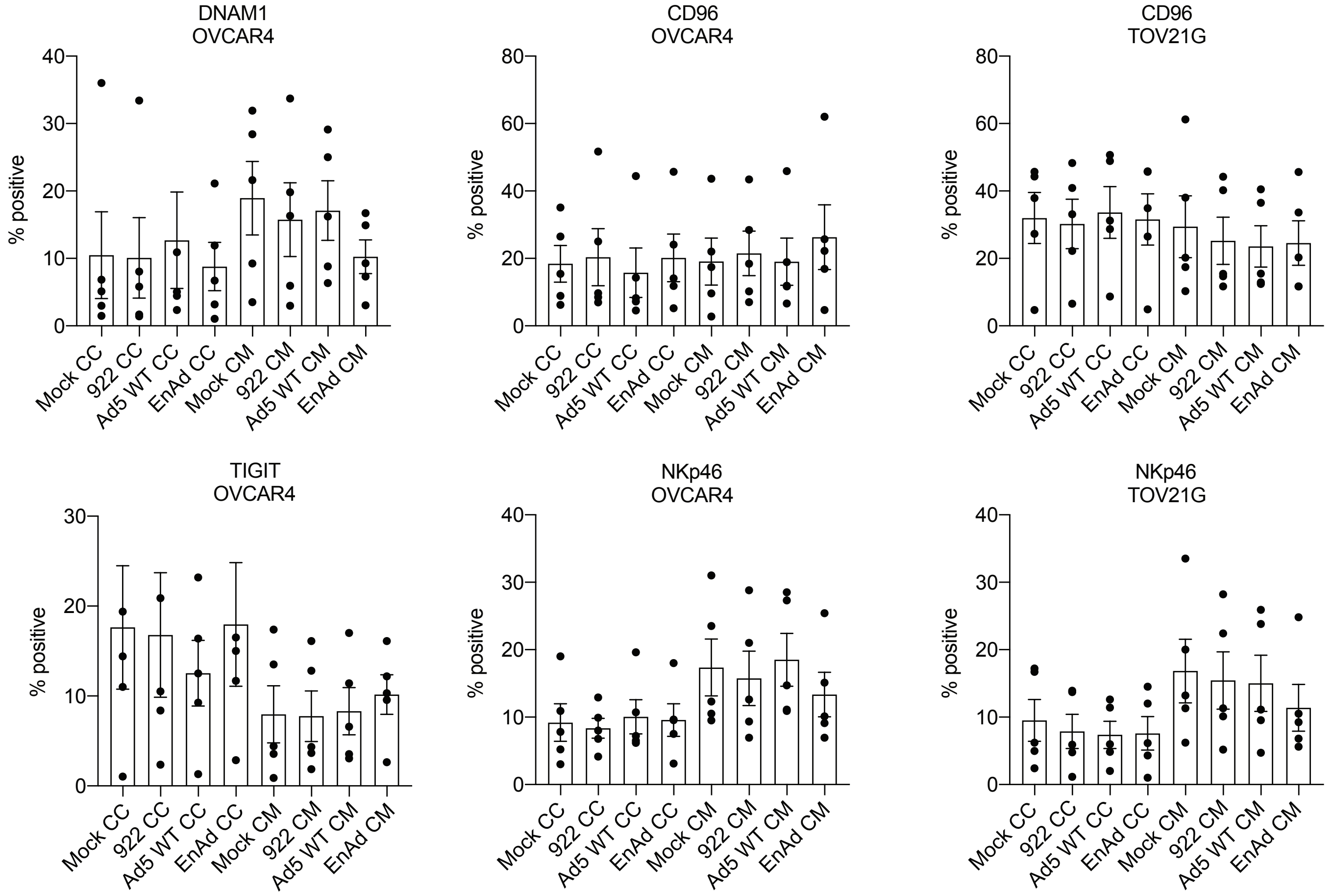

S8

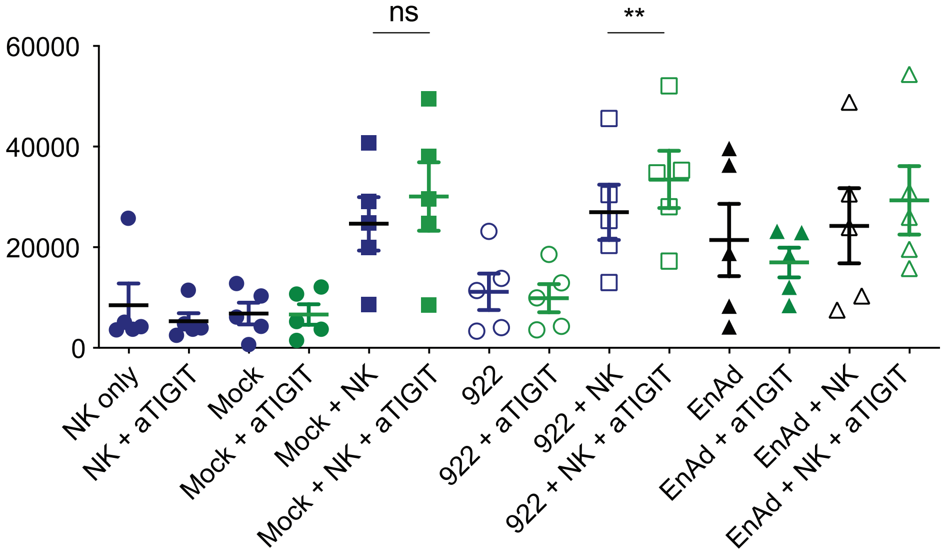

S9

```
Nectin-2 134 DEGNYTC 140
Necl-5   117 DEGNYTC 123
Ad5_III  171 PEGNYSE 177
Ad5_IVa2 220 CEGNYAP 226
Ad5_V    132 DEGNPTP 138
```

S10

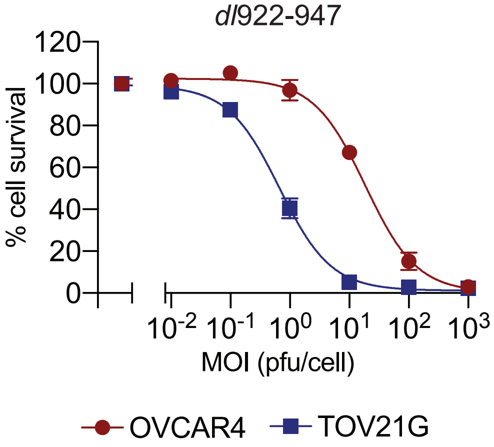

S11

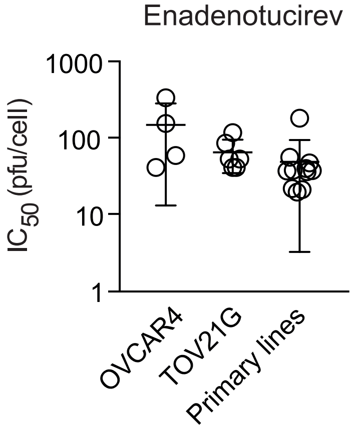

Supplement: Document S1. Figures S1–S11 [file mmc1.pdf]
